# Focused Professional Practice Evaluation and the Weaponization of Anonymous Reporting: Erosion of Care and Fairness

**DOI:** 10.7759/cureus.89056

**Published:** 2025-07-30

**Authors:** Dominic Moore, Jiawen Zhan, Ross Matlack, Mohammad Zaki, Uzma Samadani, Hamid Abbasi

**Affiliations:** 1 Spine Surgery, Inspired Spine-Avicenna Technical University (ATU), Minneapolis, USA; 2 Applied AI and Programming, Avicenna Technical University (ATU), Minneapolis, USA; 3 Healthcare Administration, Inspired Spine-Avicenna Technical University (ATU), Minneapolis, USA; 4 Legal, Avicenna Technical University (ATU), Minneapolis, USA; 5 Neurosurgery, Minneapolis Veterans Affairs Medical Centre (VAMC), Minneapolis, USA; 6 Bioinformatics and Computational Biology, University of Minnesota, Minneapolis, USA

**Keywords:** healthcare outcomes, healthcare quality and safety, healthcare systems, leadership in healthcare management and assessment of quality indicators in healthcare, patient-centred care

## Abstract

Importance

Focused professional practice evaluation (FPPE) was designed to increase patient safety, and anonymous reporting systems are often implemented to empower staff at all levels of an organization to speak up without the fear of reproach. However, the impact of these systems on patient outcomes remains largely unexamined. In this study, we survey physicians to assess whether they believe these mechanisms improve patient care or well-being.

Objective

The goal of this study was to assess potential declines in patient safety and care due to FPPE and anonymous reporting, as well as their potential misuse, through physician participation in a 29-question survey.

Design, setting, participants

A 29-question survey was conducted from February to March 2025, targeting a national sample of 152 U.S.-based surgeons. The survey included 29 questions addressing experiences with FPPE/anonymous reporting, perceptions of fairness, career consequences, and clinical practice changes.

Main outcomes

The primary outcomes that this study hoped to measure were changes in patient care and the impacts on the lives of surgeons.

Results

Of 152 physician respondents, 19.1% stated they had never been on the receiving end of a filed report. Despite this distinction, they described negative trends in patient care as well as professional practice similar to those experienced by individuals who had been reported. Respondents identified ways in which anonymous reporting and FPPE processes potentially compromise patient care: 57% (n=68) avoided high-risk cases, 70.8% (n=85) practiced defensively, 45.5% (n=56) reported erosion of operating room camaraderie and trust, and some described overprescription of narcotics or sedatives to placate staff. Many physicians feared retaliation for raising safety concerns or confronting problematic behavior, particularly from nursing staff, leading to widespread silence. In addition, many expressed personal accounts of deteriorating work culture, ruined careers, emotional distress, and even suicidal ideation. These systems not only led to real and direct harm to patients but also held the potential to be weaponized by those in power, and many respondents who felt as such cited a lack of due process, inconsistent standards, and vague or unverifiable allegations. Supporting this, 64.2% (n=79) deemed FPPE unfair, 62.3% (n=76) cited differential treatment, and 50.8% (n=62) encountered opaque or unclear disputes. Overall, only 6% (n=6) rated FPPE as an effective and positive influence on patient outcomes, with an average rating of 2.23/5.

Conclusions and relevance

Originally intended as a quality improvement measure, FPPE and anonymous 360 reporting systems create a net decline in patient care and safety, leading to real patient harm. In addition, it is often uninfluenced by metrics such as morbidity and mortality, and many claims can be made without validity, leading to serious detriments to physician mental health, retention, and professional careers. Due to the extensive implementation of these processes across many institutions, further investigation is required to appropriately guide reform, and as such, we encourage all physicians to share their personal opinions on these systems and participate in this survey [19].

## Introduction

In 2015, the publicized arrest of neurosurgeon Christopher Duntsch, colloquially nicknamed “Dr Death,” left the medical community and public masses in shocked horror. His malicious deeds have been well documented in various media, and the impact he had upon his victims and their families cannot be understated but has been recounted time and again. What is not widely discussed after this extraordinarily unusual case is the critical shift of hospital systems into further incorporating rigorous systems with which to try and identify clinicians with poor skill sets or impairments.

Medicine has a long and complex history of review processes, and many systems have been trialed over the course of this history to try and add some level of standardization, and thus accountability, to a field that defies simple classification. The field has even gone so far as to look abroad at practices championed in aviation to try and increase degrees of safety, but the skeleton of peer review processes remained the industry champion for many years [1]. Peer reviews have been mandated by the Joint Commission on Accreditation of Healthcare Organizations (JCAHO) since 1952, but Focused Professional Practice Evaluations (FPPE) entered the medical system back in 2008 at JCAHO’s behest [2]. Many of these current systems fall under the umbrella of FPPEs, though many others focus on software that allows rapid and anonymous reporting from all levels of staff to allow concerns to be voiced without fear of reprisal, which has historically deterred many healthcare staff members around the globe [3,4]. 

The intentions behind both these systems are clearly pure, and no doubt they were designed with the hopes of professional improvement and patient safety from a variety of perspectives. The protected identity of the reporter reduced the risk of retribution by those who might wield greater power and authority in the hospital hierarchy, and FPPEs provided a formal method to evaluate based on standards of care in a non-punitive route [5]. Truly, these tools seem to have been crafted for a noble purpose, but like all instruments made by man, they can be wielded with malice as easily as benevolence.

As anonymous reporting systems have risen in popularity, it has become of great concern that compliance platforms and committees do not function as intended. Like any anonymous social media platform, individuals can write whatever it is they like, regardless of their expertise or the credibility of their claims. Some institutions that have sought to taxonomize these complaints have found that the majority are related to complaints of communication or disrespectful behavior, not patient care [6,7]. Further, the input into these platforms may willingly or unwillingly pervert the standard FPPE processes and open avenues for administrators and staff to press their own agendas. And thus, the platforms can potentially be weaponized in the same way as many failed review processes in the past [8,9]. Reports needn't even be fully validated before being acted on, and providers under investigation don’t have to be proven guilty before they experience negative effects [8].

One example of how anonymous review portals make care more unsafe is due to their impact on the use of intraoperative imaging. One surgeon reported receiving an anonymous report that stated that she obtained too many radiographic images in the operating room in order to ascertain the correct level. Such a criticism would render the surgeon less likely to order such imaging in the future and render them more likely to perform a wrong-level surgery. Conversely, that same surgeon received a report on a different patient that she failed to obtain sufficient imaging in the operating room. No further investigation was done to evaluate the morbidity of these decisions by reporters or reviewers, which in turn would have revealed excellent patient outcomes. While not a peer review in name, these styles of reports inch dangerously close to the “sham peer reviews” of recent notoriety [10].

Such weaponization can become so severe that not only does it threaten physician careers, but even patient safety can take grim turns, as hospital staff can pressure providers into issuing medications in excess of what is safe under the threat of reporting “inadequate pain management." One physician has even reported patient death as the result of another provider caving to these pressures. A review showed that 0.06-2.5% of hospitalizations result in an overdose specifically relating to opioids [11]. In situations where a physician succumbs to internal pressures, all negative outcomes, including patient death, such as one survey respondent experienced, are placed solely on the physician's shoulders, not on hospital management or nursing staff.

To this end, we proposed a 29-question survey fielded in February-March 2025 among 152 U.S. surgeons to accurately assess these possible outcomes. In addition, we hope this survey will provide a safe haven to document the overall impact of anonymous reporting and FPPE systems, whether positive or negative, on surgeon burnout, stress, and practice patterns in the context of how these would affect patient care, as the goal is still and always will be to better the lives of those we serve.

## Materials and methods

From February to March 2025, we surveyed 152 U.S. surgeons via SurveyMonkey (see Appendix, Figures 3-11 ) with a 29-question instrument, titled “Surgeon Survey: Evaluation of the Patient Safety Reporting System,” designed to assess perceptions of FPPE [19]. The survey was crafted by spinal surgeons drawing solely on their clinical experience with FPPE processes and sent out to surgeons whose addresses were in the personal email archive of one of the investigators. In addition, the survey invitation was posted on LinkedIn (California, USA) and in a spine surgery WhatsApp (California, USA) chat group containing 660 surgeon members, a majority of whom practiced outside the United States. It was also posted on an interventional radiology website. Several survey responders did ask if they could send the link to other surgeons, and were permitted to do so.

Questions (Q) 1-5 collected demographics-specialty, years of experience, hospital type, gender, and race. Q6-Q29 evaluated fairness, career impact, stress, professional relationships, and patient care effects through 18 closed-ended questions (Likert scales, multiple-choice), one multi-select question (Q26), and seven open-ended prompts. Response rates ranged from 152 (Q1) to 50 (Q29), reflecting optional skips by participants.

Quantitative data from closed-ended questions were analyzed descriptively using percentages to summarize response distributions. Chi-square tests were applied to assess the statistical significance of key categorical response patterns (e.g., fairness perceptions, burnout prevalence), suitable for the exploratory design and sample size of 152. Open-ended responses were coded thematically (e.g., “retaliation,” “care avoidance”) by a single reviewer, identifying recurring patterns directly from the text without external frameworks. No additional statistical modeling was employed, focusing on initial exploration rather than hypothesis testing. Recruitment targeted practicing surgeons through professional surgical networks, yielding a sample with 55% neurosurgeons (Q1), reflecting the investigators’ specialty focus. While surgeon-specific, findings are intended to generalize to physicians under FPPE-like systems. Ethical oversight was given by the Pearl Institutional Review Board (IRB) for the purpose of this study, and exemption was granted.

## Results

Subjective impressions

Many of the sections of this survey permitted open-answer responses, and detailed here are some notable quotations from both physicians who have been reported (123 respondents) and even those who are unreported (29 respondents), which is 19.1% of all survey takers.

Reported

In response to Q21, one surgeon stated, "Negative impact on patient outcomes. I do not dare to prioritize an emergency case if it bumps another surgeon’s scheduled case, because the anesthesiology staff will file complaints." Similar sentiments are recounted by “Always looking over their shoulder to pull a knife out of their [back]” and "I avoid patients that are moderate to high risk." Another provider noted negative fallout after filing a report, saying, "I reported and was fired so they could protect a well-funded doctor that the hospital has a stake in his I.P." 

To Q25, a physician reported that FPPE "has pushed [him] to resign [his] chairmanship and professorship and move to private practice at a physician-owned hospital where the culture is much more supportive of physicians."

Unreported

One stated in response to Q16, "I’ve witnessed negative effects of this reporting system on our overall culture within the department. Some of my partners have been reported, and it did not improve the dynamics in the department but instead worsened them." When answering Q21, another noted that the FPPE system "leads to failure to report medical errors and near misses" due to the fear of punishment.

In addition to the above quotes, Table 1 includes further representation of these subjective opinions.

**Table 1:** Here are a few more subjective impressions provided from different survey takers that have been broadly categorized to highlight three common themes of FPPE complaints: Decreased Patient Safety, Contributions to an Inequitable Work Environment, and Reports that Lack Morbidity or Validity FPPE: Focused professional practice evaluation

Decreased Patient Safety	Contributions to an Inequitable Work Environment	Reports that Lack Morbidity or Validity
“In my experience, there are some nurses who routinely refuse to provide sedation and file reports against the physicians who disagree and feel that anesthesia is overkill. But ultimately, what happens is that cases are bumped. Sometimes day after day.”	“One of my colleagues attempted suicide after being relieved of his clinical privileges without cause for over one year. The chair activates this mechanism behind closed doors and then portrays himself in public as a bystander who cannot get involved. There is a wide gap between the values that the department emulates in public and their actions.”	“Once reported, one is guilty before being proved innocent. It takes a great deal of momentum and effort to reverse a report. I served as program director and spent a great deal of time getting these issues taken of the various residents records as they were one sided and often petty.”
“No point in pointing out a deficiency. Just say, ‘please’ and ‘thank you’ and watch the patient suffer”	“I have been a long-time member of the hospital and surgery oversight committees charged with reviewing reports. When our online system was launched, the promise of anonymity was made to encourage reporting; however, reports are rarely anonymous in so far as the perspective of the reporter - as conveyed in the complaint - is often a give-away as to the individual's role, if not identity. The non-physician quality leadership tends to seize on things that are non-issues or demand "correction" for the sake of "doing something;" this can be counterproductive at times, creating new obstacles to care and practice. I have also seen physician leadership weaponize the process, encouraging reporting to create cover for outcomes that they wish to see but don't wish to own. What I have not witnessed is a system that discourages blame assessment and enhances collaboration.”	“Got reported for not caring left or right L1 in urgent spine fracture case. Chief of surgery thinks it's a big deal somehow. Now I feel I can't trust prep nurses nor hospital leadership. When I used system to report failure of OR to maintain equipment leading to negative impact on pt care (O-arm not plugged in, so battery died), I received no response.”
“No point in pointing out a deficiency. Just say, ‘please’ and ‘thank you’ and watch the patient suffer”		“The system can be weaponized and once activated, the attackers can hide behind "anonymity", QI privilege, and opaque process. There is no numerator or denominator. Once people get reported once, it perpetuates. People talk. The second or third report is confirmation of guilt. I say this all as someone who believes there is role for concept of reporting and I know there are surgeons who are challenges and may even need to be dismissed. But the system can be weaponized and there in no defense.”
“We have lost experts and we have become short staffed in critical areas. Faculty don't take on tough cases. I have this relayed to me by at least 3 faculty I was told by one colleague that I had to get off another colleague's "radar" who was coming after me. When I knew I was being "watched" (but with no concrete reporting or even discussions) it significantly altered how I operated and even how I interacted with other surgeons in double attending cases.”		
	“Nurse managers often encourage their nurses to file these reports anytime they feel like their performance is being questioned (even when they have clearly made an error)”	
“I have colleagues who are undertrained (but entitled). I have to mentor and assist them A LOT (as if they were still in training). If help or give advice and they don't agree, I am a bad mentor. If I don't help, I am a bad mentor. If I try to correct dangerous situation, they find it insulting. I refuse to allow them to make mistakes (especially when it comes to the care and safety of the patient). This is a no win situation as they complain about me throughout this reporting system and the counter complaint of their "unsafe" practices seem to be ignored.”	“Negative changes. The Doctors fear the Nurses "write ups" the nurses and other hospital workers have unions and are protected, but the physicians are not.”	“I made the decision to leave after watching colleagues I respect get attacked through this system with no support, no due process, and no accountability from the department. I did not want to work in an environment where the leadership has no accountability for this outrageous behavior.”
	“Sometimes reports can result in positive change and more open/effective communication, but for instance our residents are now very leery of "pissing off nursing" in our pediatric ICU because it seems like they (the nurses) are on a campaign to beat residents "into submission".	

Survey participants

Of 152 respondents, 55% (n=83, Q1) were neurosurgeons, and 37.8% (n=57) were other specialties; 76.7% (n=115, Q2) had more than 11 years’ experience; 56% (n=84, Q3) worked in academic hospitals, and 32% (n=48) in community hospitals; 83.8% (n=119, Q4) were male; and 68.7% (n=103, Q5) were White.

Fairness and objectivity of the system

Q10 (n=113) asked respondents to opine on the fairness of the system. Among respondents, 69.4% of physicians who have received a report (n=85) claimed that the FPPE reporting system is unfair in some capacity. Of the physicians who did not receive a report (n=28), 53.85% felt similar. 

Only 5.9% of reported physicians (n=85) and 3.7% (n=27) of unreported physicians believe that reports are completely fair (Q14). An additional 18.8% of reported and 11.1% of unreported physicians stated the system is “somewhat fair” but noted concerns about subjectivity. 

Only 27.9% (n=34, Q8) reported always being informed when a report was filed, while 29.8% (n=36, Q9) discovered later that a report had been used. Additionally, 62.3% (n=76, Q11) reported inconsistent application of the process, and 50.8% (n=62, Q14) described a lack of transparency during disputes.

Additional comments described concerns about bias and manipulation of the process (Q12), such as “Chair targets enemies” (response #27), “High relative value unit (RVU) generators never reported” (response #15), and “Sham peer review” (response #67).

Impact on physicians****

An increase in levels of stress was reported by 59.3% (n=70, Q22) of physicians; 85.6% (n=101, Q23) linked FPPE to burnout (50.8% significantly); 59.8% (n=70, Q24) considered leaving. Q25 (n=55) was grim: “Fired… career ended” (response #17), “Mental health collapse” (response #19), “Sham review ruined me” (response #55). Q6 (n=56) added: “Sham peer review ruined my career” (response #55).

Patient care and relationships****

**Figure 1:**
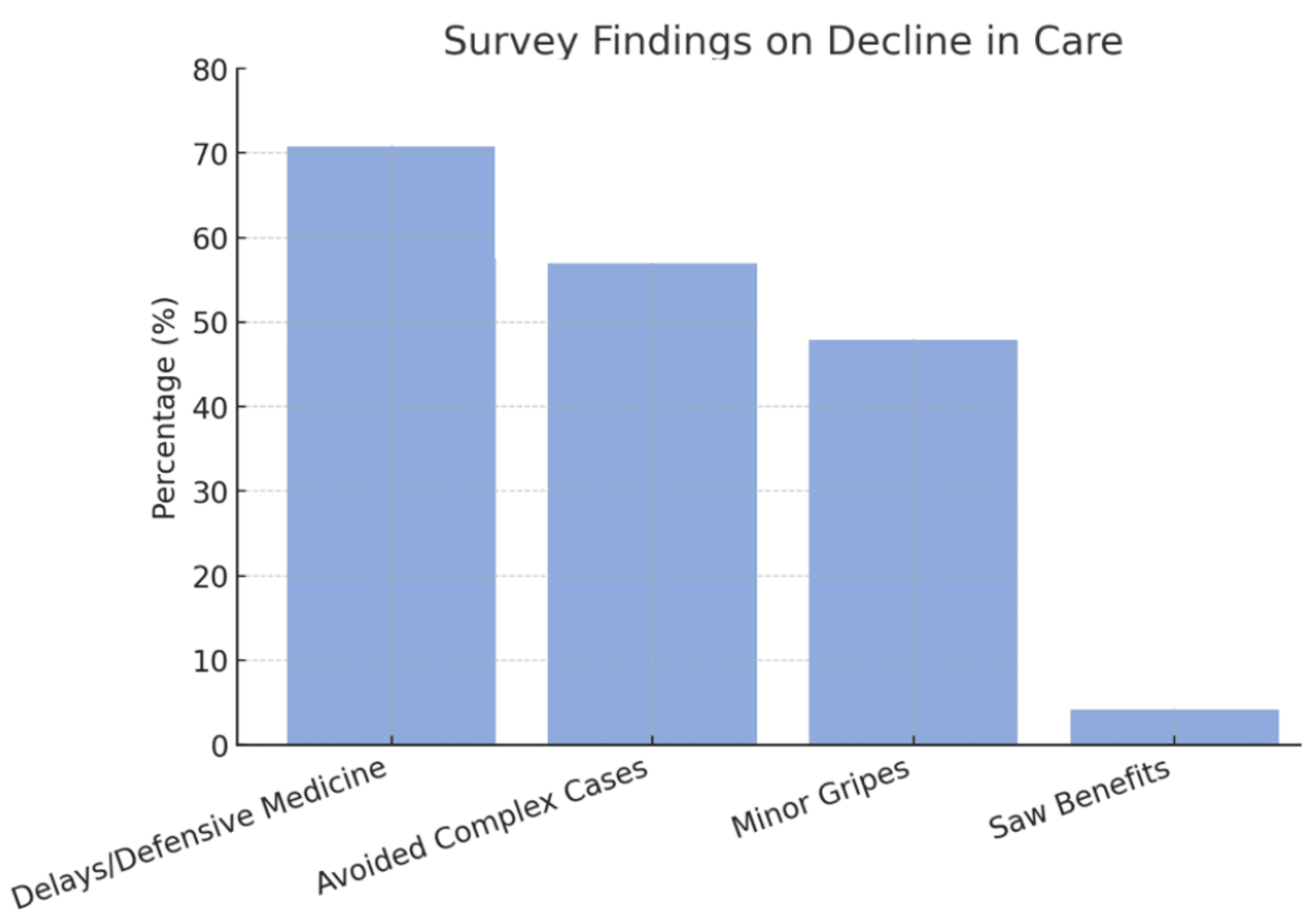
Above is a bar graph to visually display the effects of FFPE and anonymous reporting on physicians in regard to clinical practice—including delays (70.8%) and avoidance (57%)—for what nearly half of reviewers (47.9%) noted as being only minor grievances, and only 4.2% actually benefited from FPPE: Focused professional practice evaluation The image is created by the author.

Care declined, as 70.8% (n=85, Q19) of physicians noted delays/defensive medicine; 57% (n=68, Q20) avoided complex cases; only 4.2% (n=5, Q17) saw benefits, with 47.9% (n=57, Q18) flagging minor gripes. This data is better presented in Figure 1.

Q21 (n=63) warned: “High-risk patients turned away” (response #55), “No one does complex cases” (response #42). Operating room trust eroded (45.5%, n=56, Q13); 69.1% (n=85, Q15) saw blame over collaboration. Q16 (n=79) echoed: “Pits services against each other” (response #20), “No collaboration” (response #37).

Effectiveness and reform

**Figure 2:**
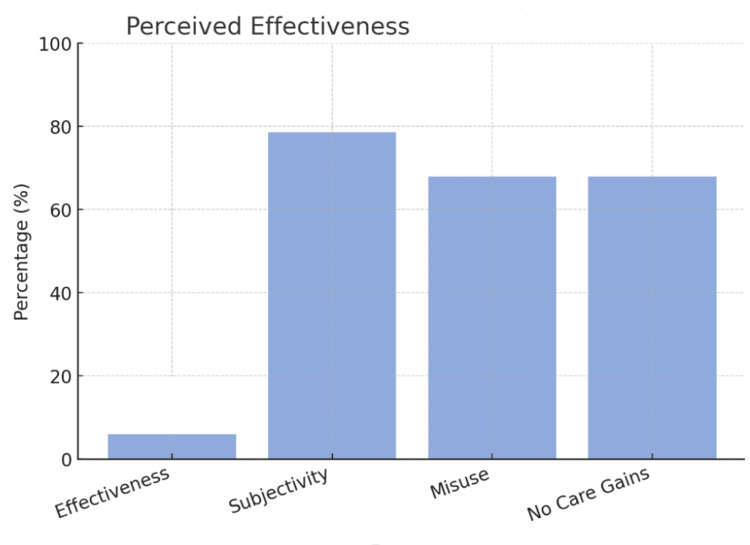
Here is shown the perceived effectiveness of this reformation process by physicians, as well as the issues raised with it. Only 6% rated it as effective, while 78.6% stated it was subjective, 67.9% that it was misused, and an equal 67.9% noting that they felt there were no appreciable care gains from the process. The image is created by the author.

**Figure 3:**
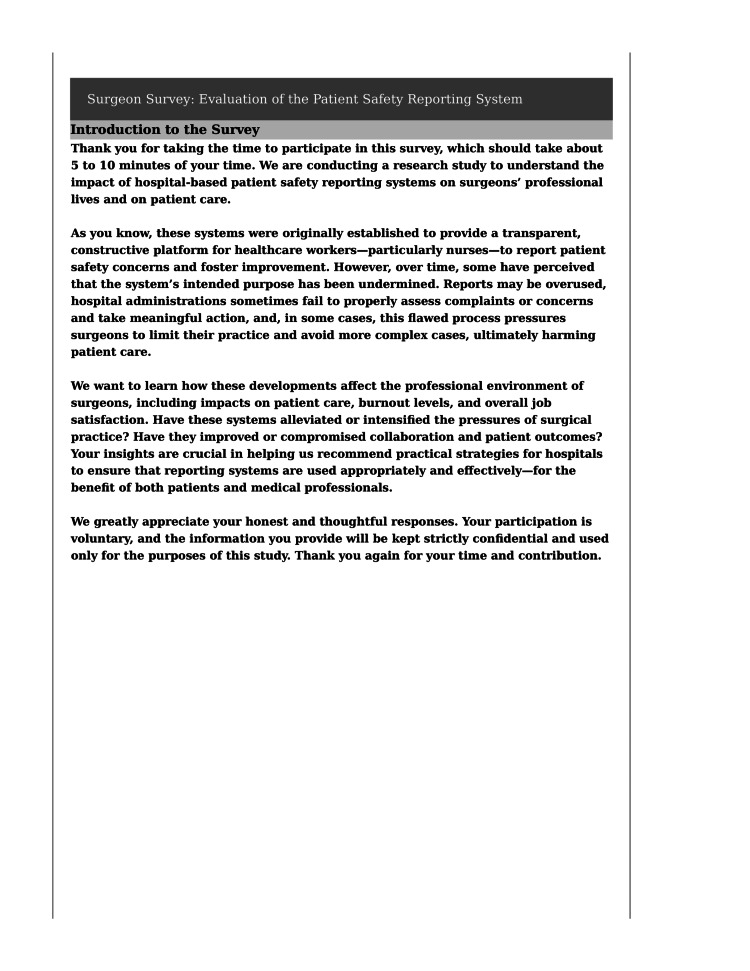
Survey page 1

**Figure 4:**
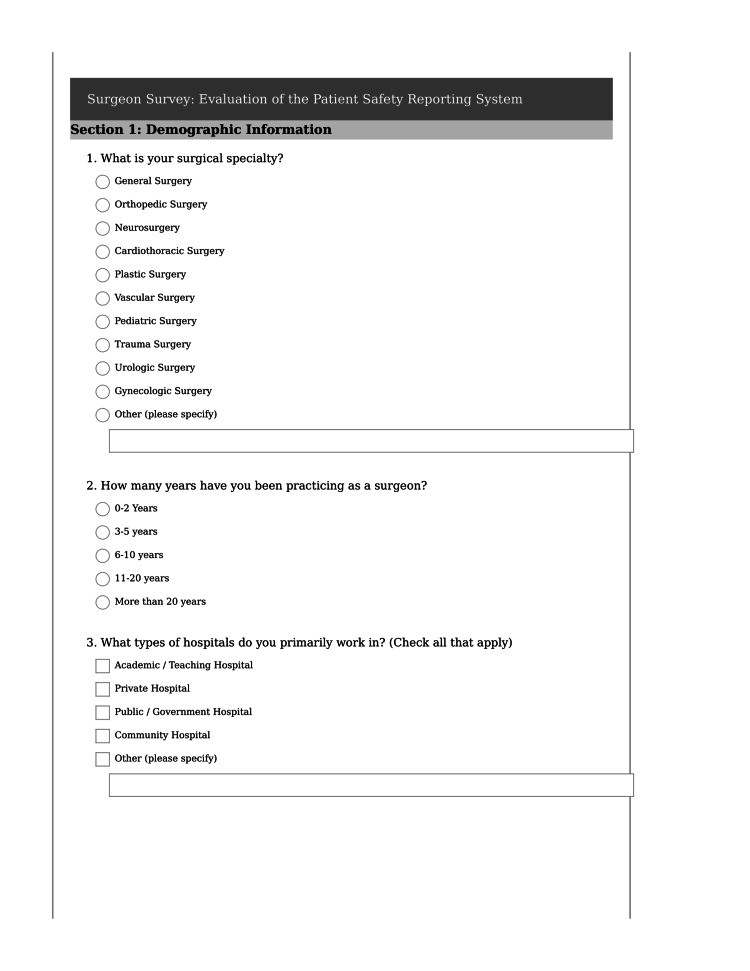
Survey page 2

**Figure 5:**
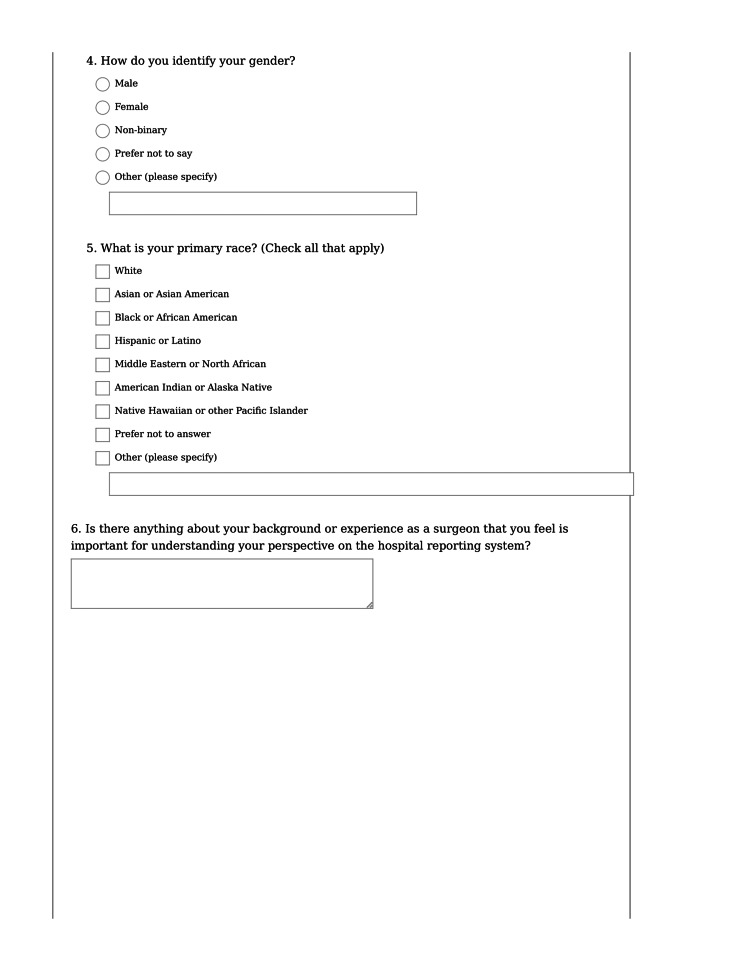
Survey page 3

**Figure 6:**
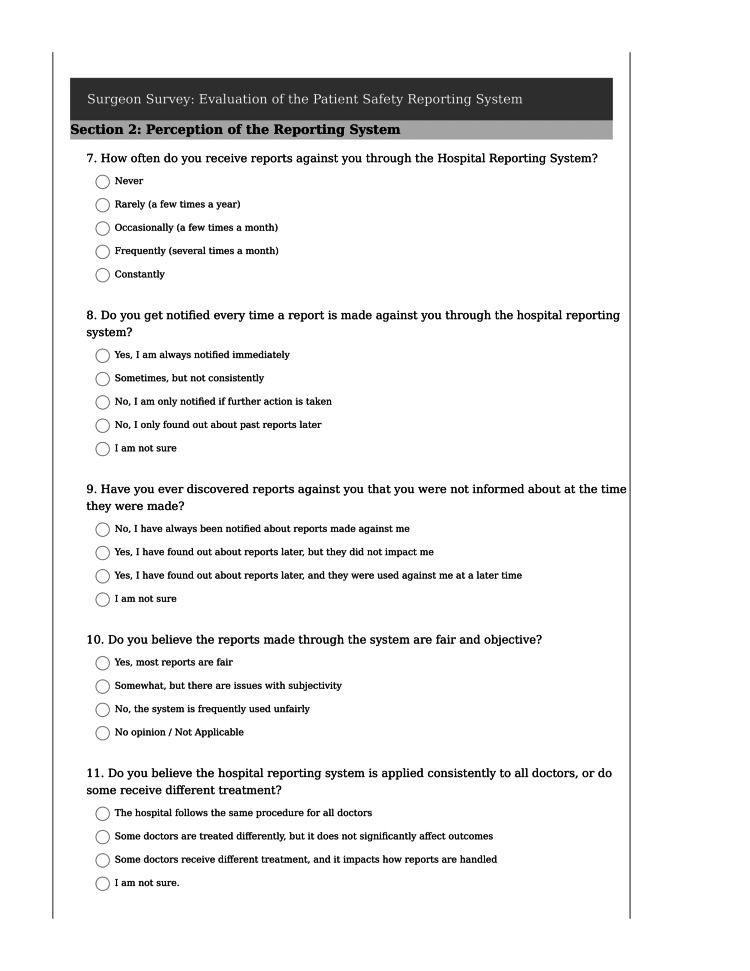
Survey page 4

**Figure 7:**
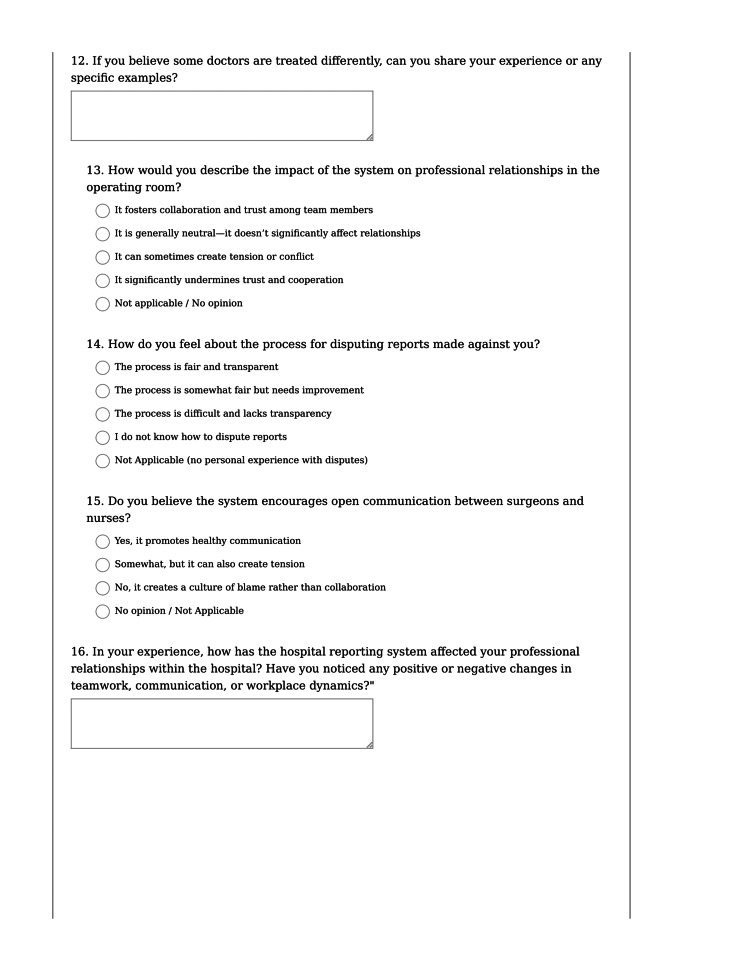
Survey page 5

**Figure 8:**
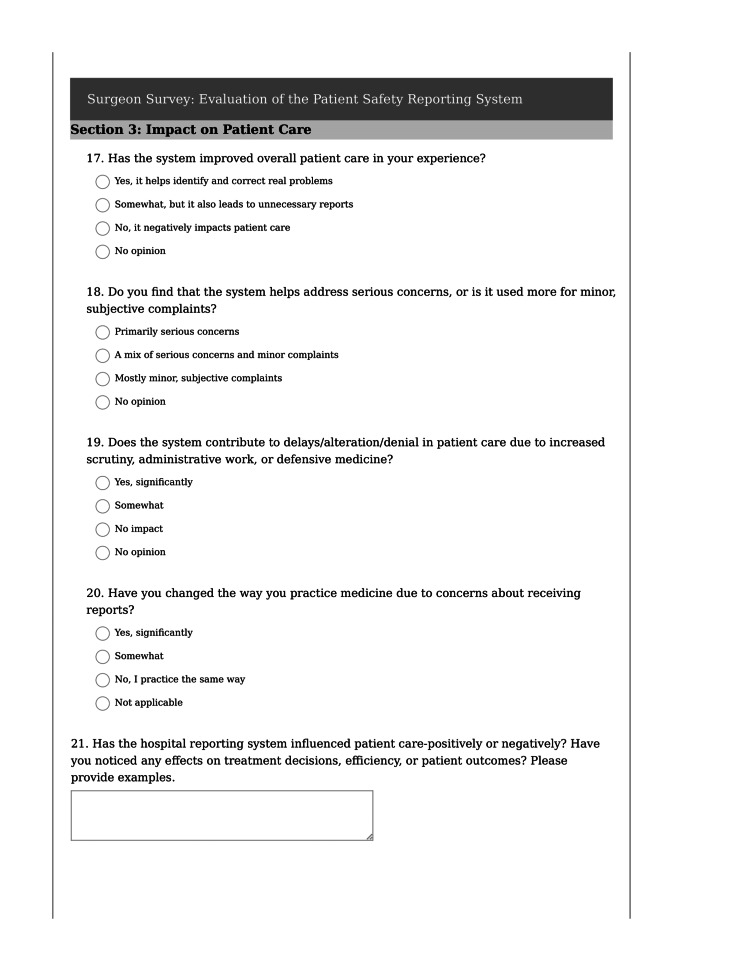
Survey page 6

**Figure 9:**
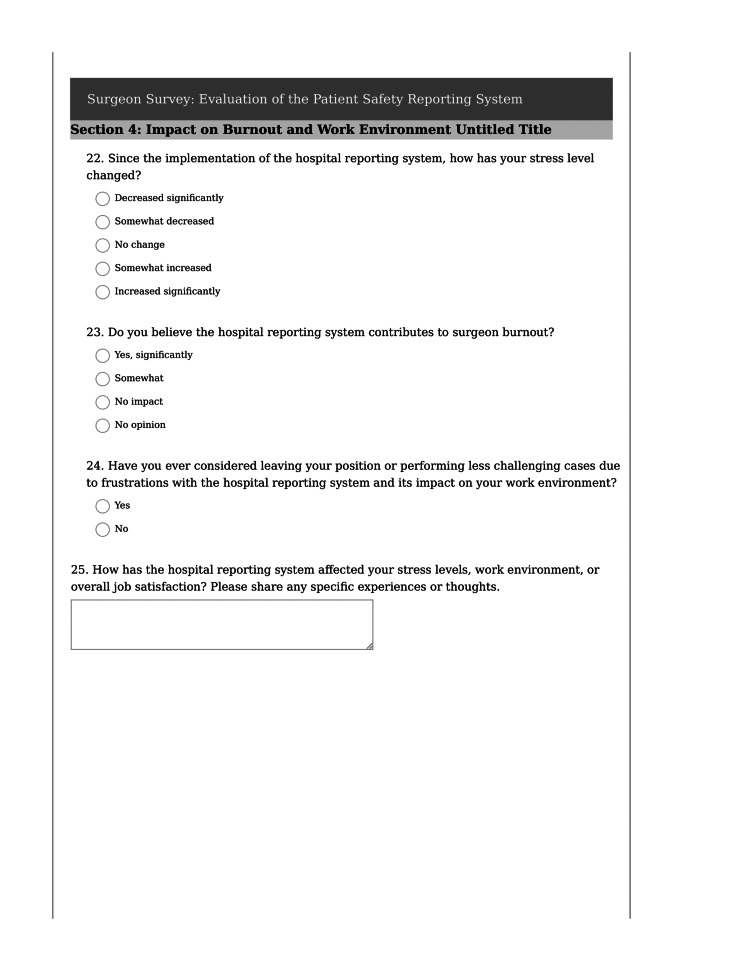
Survey page 7

**Figure 10:**
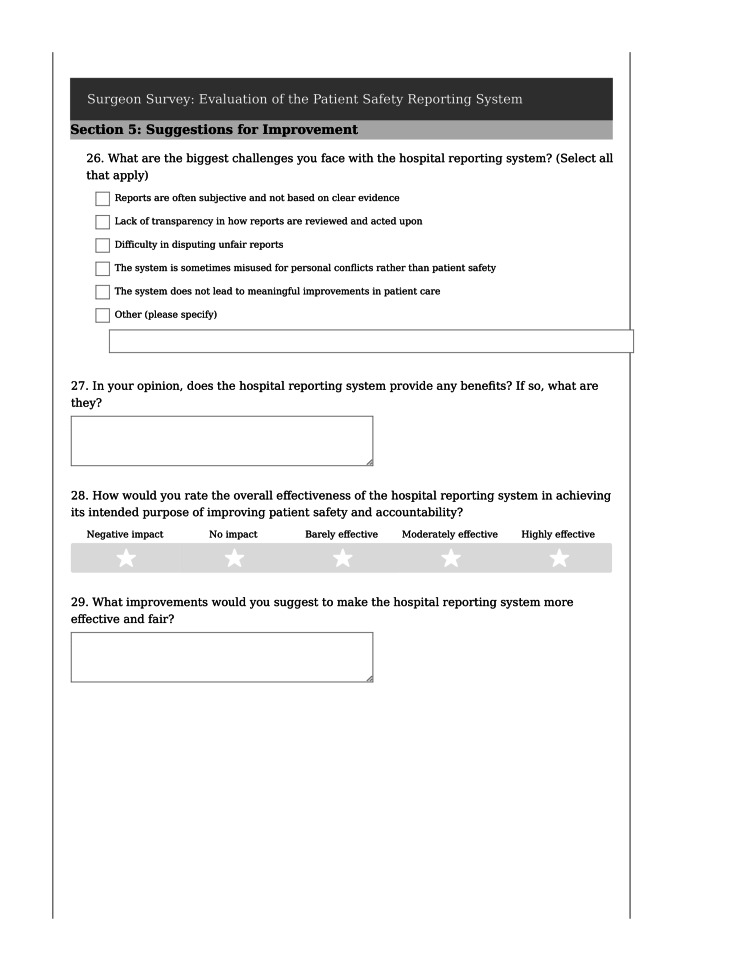
Survey page 8

**Figure 11:**
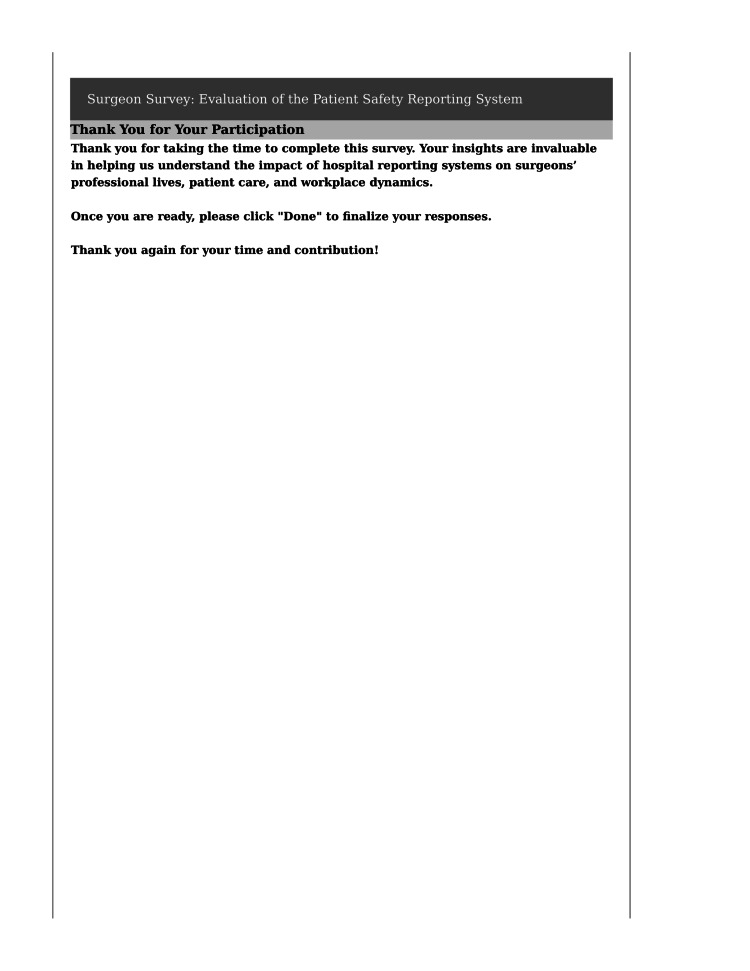
Survey page 9

FPPE combined with anonymous reporting scored 2.23/5 (Q28, n=110), with 6% (n=6) rating it effective. Challenges (Q26, n=112) included subjectivity (78.6%, n=88), misuse (67.9%, n=76), and no care gains (67.9%, n=76). Q27 (n=60) saw little upside: “No benefit” (response #9), though some noted fixes later in Q29 (n=50), where they urged transparency (response #17), non-anonymity (response #40), and care focus (response #43). This is reflected visually in Figure 2.

For swifter reference, shown below in Table 2 are some key findings on perceptions and impacts as reported by survey takers.

**Table 2:** Above are some of the key findings discovered from several multiple-choice questions in this survey. The questions are shown in the first column, the types of categories of responses represented are shown in column two, the percentages of respondents are in the third column, the chi-squared is in the fourth, and the calculated p-values are in the final column. For the purpose of this study, a p-value of 0.05 was used to determine significance, of which all listed questions other than Q14 and Q20 were shown to be significant. *Meets the threshold of <0.05 for statistical significance.

Question	Response Category	Percentage (n)	Chi-Square	p-value
Q11: Differential treatment	Yes, impacts outcomes	62.3% (76/122)	7.377	0.004191*
Q14: Dispute process fairness	Difficult, lacks transparency	50.8% (62/122)	0.0328	0.463955
Q15: Communication with nurses	No, creates blame culture	69.1% (85/123)	17.9593	1.40E-05*
Q19: Defensive medicine/delays	Yes	70.8% (85/120)	20.8333	3.00E-06*
Q20: Practice changes	Yes, avoid complex cases	57.0% (68/120)	2.1333	0.085323
Q23: Burnout contribution	Yes (significant + somewhat)	85.6% (101/118)	59.7966	0*
Q24: Consider leaving/simplifying	Yes	59.8% (70/117)	4.5214	0.02075*
Q28: Effectiveness (mean 2.23/5)	Barely effective or worse (≤2)	80.9% (89/110)	42.0364	0*

## Discussion

The overall goal of this survey was to investigate the relationship of possible reductions in patient safety due to anonymous reporting and FPPE processes, as experienced by physicians. This study reveals the perceived pitfalls of anonymous reporting and FPPE’s transformation into a retaliatory instrument, a threat not confined to surgeons but pervasive across medicine. From the displayed subjective quotes, it’s clear that the negative effects of these 360-style review systems are not isolated to those who have been directly spurned. 19.1% of respondents had never received a report, and yet many of them still shared some of the strongest impressions of the system, including quotes that detail colleague suicide attempts, resident disillusionment with medicine, and the decline of both teamwork and patient safety. Many note that these systems have a place in the review process but are widely abused, leading to worse patient care and inequity in the work environment. Many of these reports don’t even display events that lead to negative outcomes or had the reasonable potential to do so, as shown by the anecdote noted in the intro and the quotes in column three of Table 1. 

This is a significant shift in physician opinion regarding FPPEs over the last decade, as a study between 2004 and 2014 found that 86% of physician feedback regarding systems of this type was positive, so long as feedback was accurate and detailed [12]. These systems fail in this regard, as our 50.8% opaque disputes (Q14) suggest, and only 5.9% of responding physicians seem to feel that these FPPE systems meet a standard of fairness. It is unsurprising that there would be such concerns in regard to systems that have no accountability for reports, inaccurate reporting, and lack concrete requirements for validation, aside from what leaders and administrators may choose to verify or bury as they see fit. 

This narrative is enriched by highlighting cognitive biases, such as hindsight. A 2019 study on hindsight bias shows that physicians are far more critical of clinical decisions when the outcome is known compared to when it has yet to be determined, which aligns with FPPE’s 78.6% subjectivity (Q26) and 67.9% misuse [13]. The next generation of attendings is already being taught to fear the system, as shown by the quote in Table 1. When partnered with 85.6% linking FPPE to burnout (Q23) and 59.8% contemplating exit (Q24), this is yet another factor bleeding the industry into workforce anemia that has already raised alarms within the American Medical Association [14]. Respondents clearly perceive that patient care suffers as a result; 70.8% report defensive shifts (Q19) and 57% avoid complex cases (Q20), risking access gaps (Doe, 2024), as Q21’s “No one does complex cases” (response #42) warns. FPPE’s dismal 6% effectiveness (Q28) underscores a failure not worth the cost.

Professional relationships fracture, whether the recipient has received a report or not, as shown by the contradicting FPPE’s quality intent, as Q16’s “Pits services against each other” (response #20) illustrates. The majority of survey takers, reported and otherwise, note that the FPPE system has had an overall negative effect on culture and practice. Physicians so fear retaliation for speaking out that the American Medical Association has been tasked with crafting a legislative template that will help prevent them from facing retaliation for reporting such negative impacts [15]. There is an ironic twist in the fact that systems designed to promote fair reviews and anonymous reporting, free from fear of retribution, have instead shifted the burden of fear upward. Bader et al.’s call for bias mitigation aligns with Q29’s pleas for transparency (response #17) and due process (response #37), echoing Vyas & Hozain’s standardization push [16]. Though neurosurgery-heavy (55%, Q1), this surgeon-focused data reflects a physician-wide threat, necessitating a systemic reform.

Due to the evidence of reported dissatisfaction, harm, and even potentially malicious intent, it is important that we are transparent about this study’s limitations. Since this study is based on self-reported perceptions, the accounts and statistics reflect physician experiences and encounters rather than documented instances of misuse. As a physician-based study, it also does not account for the opinions of other medical staff regarding the system and any potential benefits or detriments that they see. The survey relies on convenience sampling, and as such, while physician concern is clearly documented, broader conclusions about systemic misuse require future corroboration. Additionally, studies of this breed struggle to distinguish between valid reporting of physicians that ends up being negative versus truly weaponized or retaliatory. Both sides of this discussion lack objective avenues for validating or dismissing the concerns posed in this paper, so categorization and quantification of subjective information are relied upon.

Also, given the nature of the survey, it is possible that physicians who feel targeted were more likely to respond, but to this point, it is imperative to examine the responses of those who were never reported (19.1% of respondents), which largely align with those who were reported. The distribution of survey responses leans heavily towards physicians with a high level of experience, potentially because the email archives used to initially distribute the survey contained mostly experienced surgeons, and so does not inherently reflect a disinterest in the topic from more junior surgeons. There is also no way to verify if physicians not directly employed by a hospital receive a higher proportion of reports.

The goal of these systems is to provide a venue for honest, constructive, and accurate feedback to encourage better practice and patient safety without fear of retaliation. This has not been the result, and much like the tech industry faced when implementing similar systems, healthcare finds itself staggering under the weight of reports that lack accountability, credibility, or even positive intentions [17]. This is particularly disheartening, as studies show that as many as 65% of employees at large desire feedback, but the only system currently available displays apparent signs of weaponization for unjust cause [18].

Though the purpose of this study was to determine physician' opinions on the quality of the review system that holds career-altering sway over their practices, there were some common recommendations made in regard to reform (Q29). They centered around increasing objectivity and fairness, with one recommending “a multi-disciplinary review panel that vets the reports for frivolousness prior to being sent to six different groups for review.” Some respondents desired deanonymization entirely in order to encourage greater accountability for claims, though such a choice could lead to an increase in retaliation, so the authors of this study would not recommend it lightly and suggest that it be an open topic of discussion. The potential of implementing blinded artificial intelligence into review models may also be worth considering. A larger study is desired with a wider survey population to further investigate these trends of opinion and how they might differ or align with further input.

No matter what change results from the opening of a dialogue on these systems, actionable change is necessary to protect patients, practitioners, and even hospitals that currently fail to adjudicate these current reports and open themselves up for legal retaliation. 

## Conclusions

FPPE and anonymous reporting systems, designed to enhance practice and reduce retaliation, have devolved into closed-door decision-making processes fueled by anyone without concern for accountability. This is not always problematic, and indeed, a good number of administrators likely hold true to the benevolent ideals under which these systems were conceived, but the opaque nature of many of these processes makes it all too easy for others to weaponize them, harming physicians and patient care. Our 152-surgeon survey-64.2% unfairness, 85.6% burnout, 70.8% care shifts, confirms a crisis. The FPPE system with Health Care Quality Improvement Act (HCQIA) support is important in many regards, but reform is urgently needed to restore quality across medicine and prevent further harm to an already diminishing and overworked profession. While this survey highlights strong concerns, it should serve as a starting point for further inquiry and multi-stakeholder dialogue. To this end, further research with additional perspectives is required, and anyone interested in contributing to this effort may voice their opinions, positive or negative, through this survey [19].
